# Health care seeking patterns and out of pocket payments for children under five years of age living in *Katchi Abadis* (slums), in Islamabad, Pakistan

**DOI:** 10.1186/1475-9276-13-30

**Published:** 2014-04-16

**Authors:** Aneeqa Rehman, Babar Tasneem Shaikh, Katrina A Ronis

**Affiliations:** 1 Health Systems & Policy Department, Health Services Academy, Park Road, Islamabad, Chak Shahzad 44000, Pakistan

**Keywords:** Health seeking behavior, Out of pocket payment, Under 5 children, Katchi Abadis

## Abstract

**Background:**

Since 1990, Pakistan has faced an unprecedented rate of urbanization, thereby resulting in the uncontrolled proliferation of slums (*Katchi Abadis*) in all large cities. These areas lack the basic municipal services such as safe water supply, sanitation and waste collection. There is limited access to quality health care services, both curative and preventive. Therefore, communities living in *katchi abadis* are faced with health seeking challenges and catastrophic expenditure to pay for health care services (formal or informal).

**Methods:**

This cross-sectional quantitative study was conducted in Islamabad, using a semi-structured questionnaire with mothers of children 5 years of age. There are 34 *katchi abadis*, 11 are recognized by the Capital Development Authority, out of which seven were included in the study. The calculated sample size was 207.

**Main findings:**

Average household income was found to be Pak Rupee 10,000 (approx.US$100) per month. Diarrhea, fever, common cold and cough were common illnesses among under 5 children. Approximately 43% of the mothers were illiterate and they preferred consulting a private doctor or a private dispenser in the *katchi abadi*. Mother’s level of education was significantly associated with the type of health provider consulted. Majority had to spend out of pocket, while many either borrowed money from relatives or friends or sold a household item. Delay in seeking health care added to the out of pocket expense. The mean cost on child’s treatment was approximately PkRs400 (approximately US$ 4) for a single consultation.

**Conclusion:**

There are several factors associated with health seeking behavior of mothers of children under the age of 5 years, living in the *katchi abadis* of Islamabad. The latter population group is one of the most vulnerable given their poor standard of living conditions. A multi-sectoral approach is needed to address the provision of basic amenities, the availability of safety nets to pay for health care is crucial to avoid catastrophic expenditure and the provision of community-based health promotion programs are essential to improve health seeking behaviors whilst simultaneously promoting and protecting health.

## Background

Many developing countries such as Pakistan struggle with low budget allocations to the health sector. In a low resource setting, the public health sector faces many challenges to provide quality health care to a variety of consumers. The private sector, which is perceived by some to be of superior quality, involves large out of pocket (OOP) expenditure [1], however the public sector also incurs a cost which for many is unaffordable Both the private and the public sector health care cost is pushing vulnerable population groups further into a poverty trap. The direct payments, (public or private) act as a barrier for poor people from seeking appropriate health care as early as possible. These vulnerable population groups forced to sell assets or borrow money to meet the catastrophic expenditure of health care i.e. spending a high proportion of non-subsistence expenditure on health [2,3].

In developing countries, the poverty paradigm is shifting from rural to urban areas, the growth of over-crowded slums and shanty towns, characterized by unhygienic environmental conditions (e.g. uncollected waste, unsafe water, poor drainage and open sewers), expose the communities to various health problems. As a result of poor living conditions, the child morbidity and mortality rates are many times higher in slums compared to more privileged urban neighbourhoods; some rural settlements have better health indicators [4-6]. Decisions regarding health seeking patterns are dependent upon the type of illness and the cost of available services. For example, research suggests that in South Asia, health care seeking behaviour is low when consulting a formal health facility or a trained health care provider [7,8]. Alleviation of financial barriers is a top priority for policy makers to reduce child morbidity and mortality, due to the fact that financial barriers influence the health seeking behaviours of poor communities globally [9-14]. There are many factors involved that add to the cost of health care services incurred, and thus delay in treatment. Lack of trust and non-responsiveness on the first level care centers, and secondary and tertiary government hospitals compel the parents of under five children to bypass the government’s primary health care services and go for expensive private hospitals, despite additional costs and time consumed in treatment [15,16].

Pakistan’s health system faces many challenges to improve the health indicators of its population. The poorly subsidized non-responsive public sector health system forces people to go for health care in the private sector, both formal and non-formal. Since the private sector primarily operates for profit, citizens have to pay large fees for consultations as well as for medicines, even for minor ailments [17]. In 2011–2012, the outlay of GDP for health was only 0.27% [18]; which is lower than all previous allocations [19]. This has resulted in 86.3%84.48% OOP health expenditure, which is high by world standards, but also in the Eastern Mediterranean region (Table 1) [20]. Total health expenditures in Pakistan, 25.1% are made by general government. The private expenditures constitute 72% of total health expenditures in Pakistan, out of which 92% are households’ out-of-pocket (OOP) health expenditures. Development partners and donor organizations have 3% share in total health expenditures [21]. There is no social health insurance system in the country and with rising poverty, many households are pushed to the level of catastrophic expenditure while utilizing healthcare [22].

**Table 1:** Health expenditures: globally, EMRO and Pakistan, 2009

**WHO worldwide statistics**	Global 2009*	EMRO countries 2009^*^	Pakistan 2011-2012**
Total expenditure on health as % age of GDP	8.5	4.2	0.27
Private expenditure on health as % age of total expenditure on health	38.4	46.8	72
OOP expenditure as % age of total expenditure on health	50.7	88.9	92

*World Health Statistics 2012; **Pakistan Economic Survey 2011–12.

Women and children under five years of age are the most susceptible to illnesses, and therefore need special and urgent medical attention. Since 80% of the people rely on private consultations at the first level of care in Pakistan, they ought to spend money for paying off the fee of doctor and for buying the medicines [23]. There are some regions were the social mobility of women is limited, and in combination with lower socioeconomic status living conditions, this leads to delayed medical consultation, and in some circumstances more expenses [24]. It is well established that household income is one of the major determinants in health care seeking behaviours [25,26]_._ There is a growing need to understand the broader determinants of health seeking behaviour to support and strengthen health systems within the local cultural context [27].

### Methodology

a) Study area profile

In Islamabad, there are 34 *katchi abadis*, 11 are recognized by the Capital Development Authority (CDA). The total population of slums in Islamabad is 88,437. Seven *katchi abadis* with CDA allotted house numbers were included in the study [28].

b) Study design

The study used a quasi-experimental design, using a cross-sectional community based survey, with a quantitative approach.

c) Sampling technique

Multistage sampling technique was utilised. In the first stage, legal slums (*katchi abadis*) in Islamabad were chosen as the study area, considering constraints such as time and resources, as per the sampling frame of CDA. In the second stage, proportional allocation of the calculated sample size to the identified slums was undertaken. In the third and last stage, simple random sampling using CDA sampling frame was undertaken considering our inclusion and exclusion criteria. Study participants were selected using simple random table, thereby giving them equal probability of selection. In this way, the size allocated to each of the selected *katchi abadis* was chosen. Use of simple random sampling reduced the chances of known and unknown biases.

d) Study population and selection criteria

Those households with a child under the age of five years were selected from the identified slums, and from those households where a child under-5 years of age had been sick and treated in the last month. Households having an acutely sick child were excluded from the study.

e) Sample size

Sample size was calculated by using the formula for proportion: n = $\frac{z^{2}\mathrm{pq}}{d^{2}}$; which is valid where z^2^ = the abscissa of the normal curve that cuts off an area α at the tails (1 - α equals the desired confidence level, e.g., 95%), we took it 1.96; d = is the desired level of precision, i.e. 0.05; p = the estimated proportion of an attribute that is present in the population; and q =1-p. The value for 'z’ is found in statistical tables which contain the area under the normal curve. According to World Bank 2011 OOP payment in Pakistan is 86.3% [20]. The minimum sample size calculated is 207. Allowance for non-response was kept at 10% and was utilized during data collection. There was an ethical pressure too as some of the respondents conveyed the message to other households in vicinity and they wanted to respond to this research. A total of 252 respondents were included in the study and there were no refusals per se (Table 2).

f) Data collection

A semi-structured pre-coded questionnaire was used for data collection. This tool was developed after an extensive literature review on the health seeking behaviours and health services utilization trends in Pakistan, and some regional countries. Questionnaire and consent forms were translated in Urdu, before data collection commenced. The questionnaire was piloted and amended to ensure the questions were comprehensible. Questions were asked regarding the child’s last illness or symptoms for which a treatment was sought; type of health care provider consulted; consultation fee; cost of medicines; distance to the health facility visited; cost of transport (if any); mode of arrangement of money; any budget set aside by the family for health; and willingness to participate in a health insurance program (if one introduced). This study was conducted from April to July 2012.

g) Data analysis

SPSS version 16.0 was used for the descriptive analysis and the inferential analysis was carried out using chi-square test with cross-tabulation to assess any significant associations.

h) Ethical considerations

This research was approved by the Institutional Review Board of the Health Services Academy, Islamabad. Verbal informed consent was obtained from the study participants after the researcher explained the purpose of the research. Anonymity and confidentiality of the study participants was maintained throughout the study. Dignity and respect for all the study participants was adhered to, throughout the research.

**Table 2:** Sample selected from different slums, proportionate to their population

**Slum areas of Islamabad**	**Frequency**	**Percent**
Diplomatic enclave, Muslim Colony	56	22.2%
G-8/1, Charles- Hansa Colony	51	20.2%
G-7/2, Near 66 Quarters	48	19.0%
G-7/1, Faisal Colony	27	11%
F-7/4, France Colony	35	13.9%
F-6/2, Near 100 Quarters	25	9.9%
G-7/3, Allama Iqbal Colony	10	4%
**Total**	**252**	**100**%

## Results

### Socio-demographic characteristics

The mean age of the respondents (primarily mothers of under 5 children) was 31±SD 3 years. Approximately 72% of the study participants earned a monthly income ranging between PkRs7000-15000 (US$65-145) per month. Fifty seven per cent (57%) of the study participants were employed in temporary private jobs. Forty three per cent of the women had no education and 37% had primary level education. Two thirds (61%) of the women had up to 4 children.

### Illness reported among U5 children

The reported illnesses for the under five year old children was as follows: Diarrhoea (35%, fever of undiagnosed origin (34%) common cold (11%) and gastro-intestinal disorders (8%).

### Type of health care provider consulted and why?

**Figure 1:**
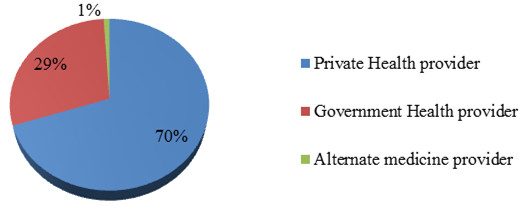
Type of health care provider consulted for treating under 5 children’s illnesses.

The majority of study participants consulted a private health care provider, either a modern allopathic doctor or a dispenser (Figure 1). Government health care providers include doctors, dispensers and lady health workers (who provided home based primary health care). The reasons for choosing the health care provider varied largely for two types of health care providers consulted by the community. Local community consulted private doctors because of the proximity (92%), their empathetic attitude (90%) and the satisfaction with the treatment provided by them (64%). Government health care providers who provide free consultation are consulted by 73% of the patients, and around one third of the mothers (33%) expressed their satisfaction with the treatment from a government doctor, dispenser or a lady health worker. Data reveals that a significant association between the place of residence and type of health care provider consulted (p = 0.00). Mother’s educational status was also found to be strongly associated with type of provider consulted for U5 child’s illness (p = 0.01).

### Expenses incurred on treating the illness of U5 children and OOP trend

**Figure 2:**
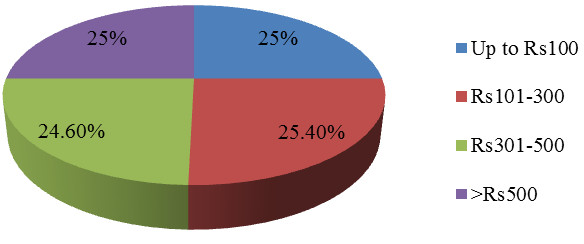
Expenses incurred for treating under 5 children’s illnesses.

The mean expense incurred on treatment for children under the age of five years and their illnesses was PkRs400 (approximately US$4), though it ranges from PkRs100 to more than PkRs500 (as shown in Figure 2). A large majority (86%) of the study participants had to bear this expense out of pocket, either by borrowing money from a neighbour or a relative (42%); or by selling their household belongings (23%).

Data shows strong statistical association between arrangement of money spent on U5 child’s treatment and immediate consultation with the health provider (p = 0.00). Strong or significant statistical association was found between total expense on illness of under five children and type of health care provider (p = 0.00); where private providers’ treatment was far more expensive. No significant statistical association was found between the expenses incurred and household income, place of residence, and gender of the sick child. When asked about the saving practices and willingness to put aside some money for such hard times, almost none of the study respondents reported on keeping such savings; however 48% showed their willingness to join a saving scheme, if introduced in their area.

### Suggestions by mothers for the better healthcare of U5 children

Thirty seven per cent (37%) of the study participants demanded or requested that a qualified doctor be posted in their vicinity on a full time basis. Free or subsidized medical services for children and pregnant women also emerged to be one of the demands (32%). More than one third of the study participants did not know what to suggest for improving the financial safety net of health care provision.

## Discussion

Health care seeking behaviours and patterns are dependent on a host of factors; amongst which the financial status of the household is a major determinant [29,30]. The results of this study validate the existing literature, hence reinforcing the association between family income and socio-economic status of the household with the choice of health care provider to be consulted. OOP payments were incurred by a large proportion of the study population, similar to national data [21,31]. In our study area, significant number of population lives just above the poverty line. The minimum wages declared by the Government of Pakistan are PkRs9000 (approximately US$91) per month. Living on limited budgets may have contributed to in the delay in health care seeking behaviour for the mothers of sick children [24]. Our study confirms that immediate consultation with a health care provider is strongly associated with the disposable income of the family at the time of the child’s illness.

The preference by the study participants to consult a private healthcare provider, is a major finding which reflects findings of the Pakistan Demographic & Health Survey 2007–08 [32]. Due to the fact that private medical care is relatively expensive, people believe that they should save some money for such catastrophic expenditures related to episodes of illness. Such catastrophic expenses push families into ultra-poverty conditions [33]. The Capital Administration Authority must be sensitised to such findings to plan for the provision of safety nets or a social protection mechanism to poor families of slum dwellings living in Islamabad, especially with respect to their health expenses. Empowering women through education and access to health information and great employment opportunities is an effective conduit in promoting appropriate and timely health care seeking behaviour for children [26,34]. In the absence of such interventions, there is a strong likelihood that such health inequities will further increase and will make this vulnerable population suffer even more.

## Conclusion

The high OOP expenditures (or catastrophic expenditures) on child health care seeking behaviour has serious implications in terms of pushing the disadvantaged communities of *katchi abadis* into cyclical poverty wheel. A safety net in the form of vouchers, cash transfers or community based health insurance is crucial to address such glaring inequalities and inequities in health care seeking behaviour. As an immediate intervention improving the quality of services in the government dispensaries and having a 24/7 emergency care available could reduce the financial burden on the parents of children under the age of five years, living in the fragile habitat of the *katchi abadis*.

## Competing interests

The authors declare that they have no competing interests.

## Authors’ contributions

AR conceived the study, designed methodology, performed the data collection and drafted the initial manuscript. BTS supervised the study design development, data collection, preliminary write up, and contributed to all subsequent drafts. KAR suggested on write up, syntax, revision of drafts and finalization of manuscript by adding intellectual content to the paper. All authors read and approved the final draft.
